# Effect of high-fat diet and empagliflozin on cardiac proteins in mice

**DOI:** 10.1186/s12986-022-00705-0

**Published:** 2022-10-14

**Authors:** Xiaoyu Pan, Shuchun Chen, Xing Chen, Qingjuan Ren, Lin Yue, Shu Niu, Zelin Li, Ruiyi Zhu, Xiaoyi Chen, Zhuoya Jia, Ruoxi Zhen, Jiangli Ban

**Affiliations:** 1 Department of Internal Medicine, Hebei Medical University, Shijiazhuang, China; 2 Department of Endocrinology, Hebei General Hospital, Shijiazhuang, China; 3 Department of Nephrology, Hebei General Hospital, Shijiazhuang, China

**Keywords:** Obesity, Differentially expressed proteins, Empagliflozin, Cardioprotective

## Abstract

Using proteomic techniques the impact of the sodium-glucose transport protein 2 inhibitor empagliflozin on cardiac protein expression in a mouse model was assessed under normal and high-fat diet (HFD) conditions. We examined the effect of obesity on serological markers and heart function in obese mice treated with or without empagliflozin and used proteomic techniques to investigate alterations in cardiac protein expression. Using bioinformatic techniques, data were screened for differentially expressed proteins (DEPs) implicated in the putative mechanism of empagliflozin's cardioprotective effects. In C57BL/6 mice, HFD increased body weight, blood lipid, and glucose levels and was associated with structural damage to the heart. Empagliflozin reduces body weight, improves glucose and lipid metabolism, alleviates obesity-induced cardiac ventricular wall thickening, and lowers cardiac tissue collagen. The expression of several proteins was altered in the heart, mainly related to lipid metabolism. Following empagliflozin treatment, the expression of several lipid metabolism-related proteins was considerably reduced. Further examination of DEPs revealed that following empagliflozin treatment, the expressions of Apoe, Apoc1, Saa2, Apoa2, and Pon1 altered dramatically, suggesting that these proteins may be the main proteins that empagliflozin uses to treat obesity-induced aberrant lipid metabolism. Empagliflozin may protect the heart by altering the expression of genes including Apoe, Apoc1, Saa2, Apoa2, and Pon1, which are all involved in lipid metabolism disturbance in obesity.

**Supplementary Information:**

The online version contains supplementary material available at 10.1186/s12986-022-00705-0.

## Introduction

Sodium-glucose transport protein 2 inhibitors (SGLT2i) are new oral hypoglycemics that lower blood sugar by decreasing renal glucose reabsorption and increasing urine glucose excretion [1]. SGLT2i is useful in organic complications of diabetes, particularly cardiovascular disease [2], in addition to its hypoglycemic impact. SGLT2i has been proven in numerous studies to reduce the relative risk of cardiovascular death, all-cause mortality, and rehospitalization rates, significantly improving patient quality of life [3–5]. SGLT2i's cardioprotective impact is independent of its hypoglycemic effect, which is why it's been utilized to treat a range of different disorders, with obesity being the most common [6, 7]. By suppressing inflammation and decreasing insulin resistance, SGLT2i has been demonstrated to protect against obesity-induced heart damage [8, 9]. However, the mechanism of action for this cardioprotective effect is not well understood.

As global living standards rise, the increase in high-fat, high-energy diets has led to an increase in the prevalence of obesity [10]. Obesity-related metabolic illnesses, such as type-2 diabetes mellitus (T2DM) and cardiovascular disease (CVD), are becoming increasingly common. Obesity has been found to significantly raise the risk of CVD, increasing the incidence of cardiovascular events and worsening the prognosis in patients with pre-existing CVD [11, 12]. Obesity is thought to promote CVD as a result of chronic low-grade inflammation and insulin resistance [13].

Proteomics is a powerful tool for analyzing single proteins and complex protein mixtures. Its goal is to uncover the inherent relationships between biological genotypes and phenotypes, resulting in innovative and realistic concepts for disease diagnosis and therapy [14]. Proteomic techniques have been used to detect protein expression in animal and human heart tissue studies [15, 16]. However, most current research on cardioprotective medicines in obese patients is limited to clinical observational studies, and the drugs' mechanisms of action are unknown.

A HFD-induced obesity mouse model was constructed in this study. The trial medicine was empagliflozin, which is an SGLT2i. To investigate the mechanism of cardiac injury induced by obesity and the cardioprotective effect of empagliflozin, we employed proteomic techniques to examine cardiac protein expression in obese mice and the changes in protein expression after empagliflozin intervention.

## Methods

### Animal models and interventions

A total of 24 6-week-old C57BL/6 mice were purchased from Hebei Yiweiwo Biotechnology Co. Ltd. (Shijiazhuang, China). The mice were randomly divided into 2 groups, the control group (WC, *n* = 8) and the high-fat group (WF, *n* = 16). The WC group was given standard mouse chow (Energy supply ratio: protein 27.38%, fat 14.5%, carbohydrate 58.12%, energy value 3.48 kcal g-1) and the WF group was given high-fat chow (Energy supply ratio: protein 20%, carbohydrate 20%, fat 60%, total energy 5.24 kcal g-1). Specific diet components are listed in Additional file 1. Both were purchased from Beijing Huafukang Biotechnology Co. Ltd. (Beijing, China). The success of the obesity model was assessed by mouse weight (obese mice weighing 20% more than the control group). The WF group was then randomly divided into two groups, the WF group and the WF + empagliflozin group (WE). The WE group was given 10 mg/kg/day of empagliflozin by gavage, while the remaining two groups were given equal amounts of saline by gavage for 12 weeks. This experiment was approved by the Animal Ethics Association of the Hebei Provincial People's Hospital. All mice were supplied with abundant food and water and were housed in a temperature-controlled (22 ± 2 °C) and moderately adapted sterile animal room, which was set up in a 12 h:12 h day and night environment in order to simulate everyday life conditions.

### Echocardiography

Ultrasound examinations were performed on three groups of three mice each using a Vevo 2100 small animal ultrasound imaging system (Visualsonics, Toronto, Ontario, Canada). Animals’ chests were shaved the day before analysis. Mice were anesthetized by isoflurane inhalation, then fixed in the supine position on a 37 °C thermostatic fixation table, coated with a coupling agent, and the probe was placed on the left anterior thorax, and left ventricular motion was recorded in the short-axis view of the left ventricle using M-mode ultrasound.

### Serum sample measurement

An oral glucose load (2 g/kg dose) was administered to assess the glucose tolerance of the mice. Blood glucose was obtained at each time point (0, 15, 30, 60 and 120 min) after 6 h of fasting by measuring the tail-tip blood of mice with a glucometer and test strips (Accu-CHEK, USA). All mice were fasted for 24 h prior to blood collection and anesthetized by intraperitoneal injection of 1% sodium pentobarbital solution, and then the eyes were removed to collect blood. Blood samples were first allowed to stand at room temperature for 30 min and then centrifuged at 3500 rpm for 10 min at 4 °C. The supernatant was collected and stored at − 80 °C. Serum low-density lipoprotein cholesterol (LDL-C), high-density lipoprotein cholesterol (HDL-C), total cholesterol (TC) and triglycerides (TG) levels were measured by enzyme-labeled method and corresponding commercial kits (Nanjing Jiancheng Bioengineering Research Institute Co., Ltd., Nanjing, China).

### Tissue sample collection

Fully anesthetized mice were placed on an ice table, the thoracic cavity was rapidly opened, the peri-heart tissue was bluntly separated, the vessels were cut from the bottom of the heart, and the heart was removed and subsequently immersed in pre-chilled saline (4 °C) and the heart was gently squeezed to clean the residual blood. A portion of the heart tissue was immersed in 4% paraformaldehyde pending subsequent pathological examination, and the rest of the heart was rapidly placed in liquid nitrogen and subsequently stored in a − 80 °C refrigerator.

### Histopathological studies

Heart tissue was fixed in paraformaldehyde for at least 24 h, followed by conventional paraffin embedding and sectioning (thickness 5–6 µm). Sections were stained according to a standard staining protocol of HE, Masson's trichrome staining and Oil Red staining (http://www.servicebio.cn/). All heart sections were evaluated for histomorphology and collagen deposition using Eclipse Ci-L photomicroscopy (Nikon, Japan). Image-Pro Plus 6.0 was used for data analysis.

### Protein extraction and digestion

SDT buffer (4% SDS, 100 mM DTT, 150 mM Tris-HCl pH 8.0) was used for cardiac tissue lysis and protein extraction. Protein was quantified using BCA Protein Assay Kit (Bio-Rad, USA). Briefly, 200 μg of cardiac tissue protein was co-incubated with 30 µl of SDT buffer using UA buffer (8 M urea, 150 mM Tris-HCl pH 8.0) to remove detergents, DTT and other low molecular weight components by repeated ultrafiltration (Microcon unit, 10kD) and samples were incubated with iodoacetamide (100 mM IAA in UA buffer) for 30 min in the dark at room temperature. Trypsin digestion was performed according to the filter-assisted sample preparation (FASP) procedure described by Matthias Mann [17]. Digested peptides from each sample were desalted on C18 cartridges, concentrated by vacuum centrifugation and reconstituted in 40 µl of 0.1% (v/v) formic acid. Peptide content was estimated by UV spectral density at 280 nm.

### Peptide labelling and strong cation exchange (SCX) fractionation

The 100 μg peptide mixture extracted from each sample was labeled using iTRAQ reagent (Applied Biosystems, Toronto, Ontario, Canada) and TMT reagent (Thermo Scientific, Santa Clara, California, USA), and the operation was performed strictly according to the instructions. Fractionation of each group of labeled peptides was done by SCX chromatography on an AKTA Purifier system (GE Healthcare, Pittsburgh, Pennsylvania, USA). The peptide mixture was then reconstituted, acidified with buffer A (10 mM KH_2_PO_4_ in 25% ACN, pH 3.0), and transferred to a PolySULFOETHYL 4.6 × 100 mm columns (5 µm, 200 Å, PolyLC Inc, Columbia, Maryland, USA). Peptides were eluted at a flow rate of 1 ml/min in a gradient of buffer B. Briefly, peptides were eluted in gradients of 0%, 0–10%, 10–20%, 20–45%, 45–100% and 100% of buffer B (500 mM KCl, 10 mM KH_2_PO_4_ in 25% ACN, pH 3.0) in elution for 25 min, 25 min, 30 min, 40 min, 50 min and 60 min, respectively. Fractions were collected every 1 min and the collected fractions were desalted on a C18 column (Empore™ SPE column C18, bed size 7 mm, volume 3 ml, Sigma, St. Louis, MO, USA) and concentrated by vacuum centrifugation.

### LC–MS/MS analysis

Fractions were analyzed using a Q Exactive mass spectrometer (Thermo Scientific, Santa Clara, California, USA) was used for LC–MS/MS analysis of labeled peptides, which was coupled with Easy nLC (Thermo Fisher Scientific) for 90 min. The labeled peptides were then transferred to a reversed-phase trap column (Thermo Scientific), and a C18 reversed-phase analytical column (Thermo Scientific) in buffer A (0.1% formic acid) with a linear gradient separation in buffer B (84% acetonitrile and 0.1% formic acid) at a flow rate of 300 nl/min. Peptide identification mode was enabled, using a data-dependent top-10 method to acquire mass spectrometry data, selecting the most abundant precursor ions from the survey scan (300–1800 m/z) followed by HCD fragmentation. The resolution of the HCD spectra was set to 17,500 at m/z 200 and the isolation width was 2 m/z.

### Identification and quantitation of proteins

MS raw data obtained for each sample were searched using MASCOT (Matrix Science, London, UK; version 2.2) to filter out differentially expressed proteins (DEPs). The acquisition of DEPs between groups was screened by two factors, expression difference fold change (FC) and *P*-value (T-test), which significantly down-regulated proteins (FC < 0.83 and *P* < 0.05) and significantly up-regulated (FC > 1.2 and *P* < 0.05).

### Bioinformatic analysis

GO (Gene Ontology) and KEGG (Kyoto Encyclopedia of Genes and Genomes) enrichment analysis was performed on DEPs. GO functional annotation is divided into 3 main categories: Biological Process (BP), Molecular Function (MF) and Cellular Component (CC) to understand the function, localization and biological pathways involved in the protein of the organism. Blast2Go (https://www.blast2go.com/) software performs GO functional annotation of DEPs [18]. The KEGG pathway database (https://www.kegg.jp/) [19] is used for pathway annotation of DEPs in order to understand the pathway information involved in the protein in question. Fisher's exact test was used to derive the significance of differences between groups and thus find DEPs enrichment information (*P*-value < 0.05). The final string tool (https://cn.string-db.org/) [20] was used to analyze the interrelationships between DEPs and to construct protein–protein interaction (PPI) networks.

### Statistical processing

The experimental data were analyzed using Graphpad 8.0 software, and the results were expressed as Mean ± SD. One-way ANOVA was used for comparison between multiple groups, and LSD-t test was used for multiple comparisons (False-discovery rate ≤ 0.01). We defined *P* < 0.05 as a statistically significant difference.

## Results

### Body weight change

**Fig. 1:**
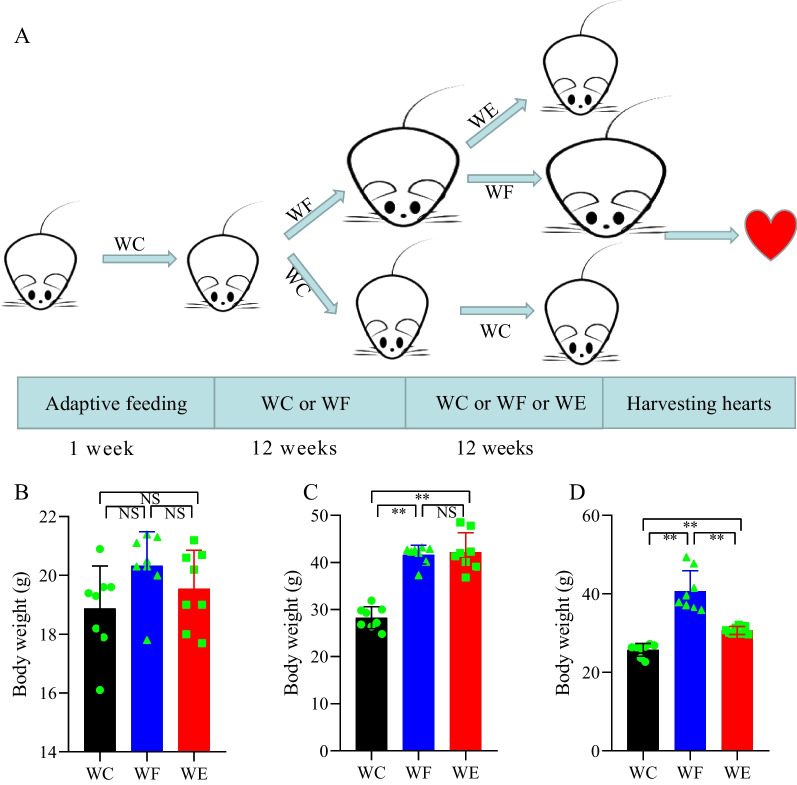
Effect of empagliflozin on body weight in obese mice. **A** Overview of the research flow chart. **B** Body weight of all mice after one week of adaptive feeding (*n* = 8/group). **C** Changes in body weight of mice after 12 weeks of high-fat diet intervention (*n* = 8/group). **D** Body weight changes in mice after 12 weeks of empagliflozin and high-fat diet intervention (*n* = 8/group). Body weight values are expressed as means ± standard deviation. NS represents *P* < 0.05, **represents *P* < 0.01. Abbreviations: NCD, normal chow diet; HFD, high fat diet; WC, control group; WF, high-fat diet; WE, high-fat diet + empagliflozin

Figure 1 demonstrates the changes in body weight of mice in each group before high-fat feeding, after high-fat feeding and after empagliflozin intervention. There was no significant difference in body weight between the groups after 1 week of adaptive feeding (*P* > 0.05). After 12 weeks of high-fat feeding, the body weight of mice in the WF group was significantly greater than that in the WC group (*P* < 0.01). The WE group was treated with empagliflozin, and after 12 weeks the body weight of the model mice decreased (24.6%) compared to the WF group, but still increased (16.1%) compared to the WC group (*P* < 0.01).

### Changes in serological indicators

**Fig. 2:**
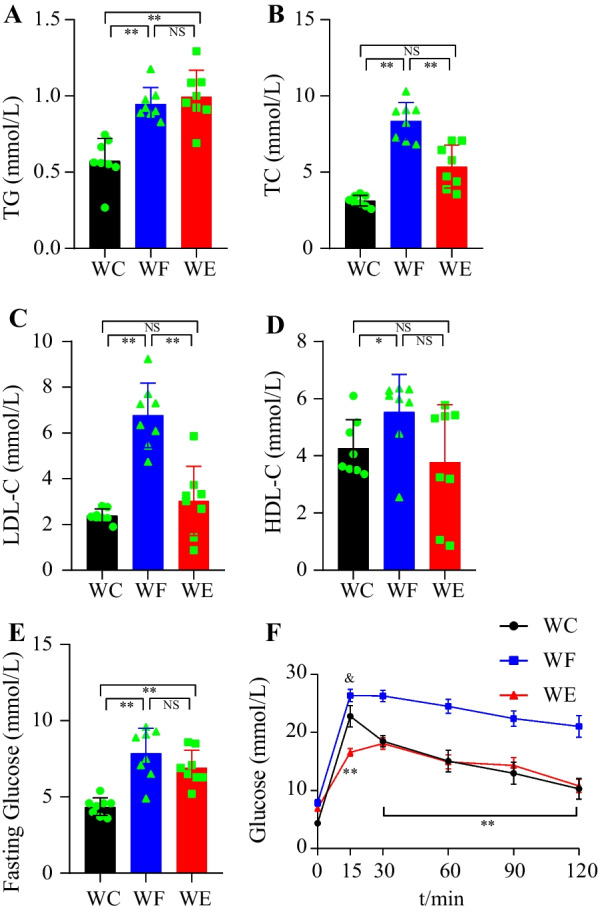
Effect of empagliflozin treatment on serological parameters and body weight. **A** TG (*n* = 8/group). **B** TC (*n* = 8/group). **C** LDL-C (*n* = 8/group). **D** HDL-C (*n* = 8/group). **E** Fasting Glucose (*n* = 8/group). **F** Glucose tolerance test curve. & indicates WC group compared with WF group, *P* < 0.05. NS represents *P* > 0.05, *represents *P* < 0.05; **represents *P* < 0.01. Abbreviations: WC, control group; WF, high-fat diet; WE, high-fat diet + empagliflozin; TC, total cholesterol; TG, total cholesterol; LDL-C, low-density lipoprotein cholesterol; HDL-C, high-density lipoprotein cholesterol

Obesity significantly increased lipid and fasting blood glucose levels. Fasting glucose, LDL-C, HDL-C, TC and TG were significantly increased in the WF group compared with the WC group (*P* < 0.05). Serum TC and LDL-C were significantly better in the WE group compared with the WF group after empagliflozin treatment (*P* < 0.01), while TG, HDL-C and fasting glucose were not significantly changed (*P* > 0.05). The changes in glucose tolerance of mice showed a significant increase in blood glucose values in all groups of mice. In the WF group, the blood glucose decreased slowly after reaching the peak, and the blood glucose values increased significantly at 15, 30, 60, 90 and 120 min compared with the WC and WE groups. Blood glucose in the WE group increased slowly and reached the peak at 30 min, after which there was no significant difference with the blood glucose in the WC group. Lipid and glucose data are shown in Fig. 2.

### Cardiac pathological changes

**Fig. 3:**
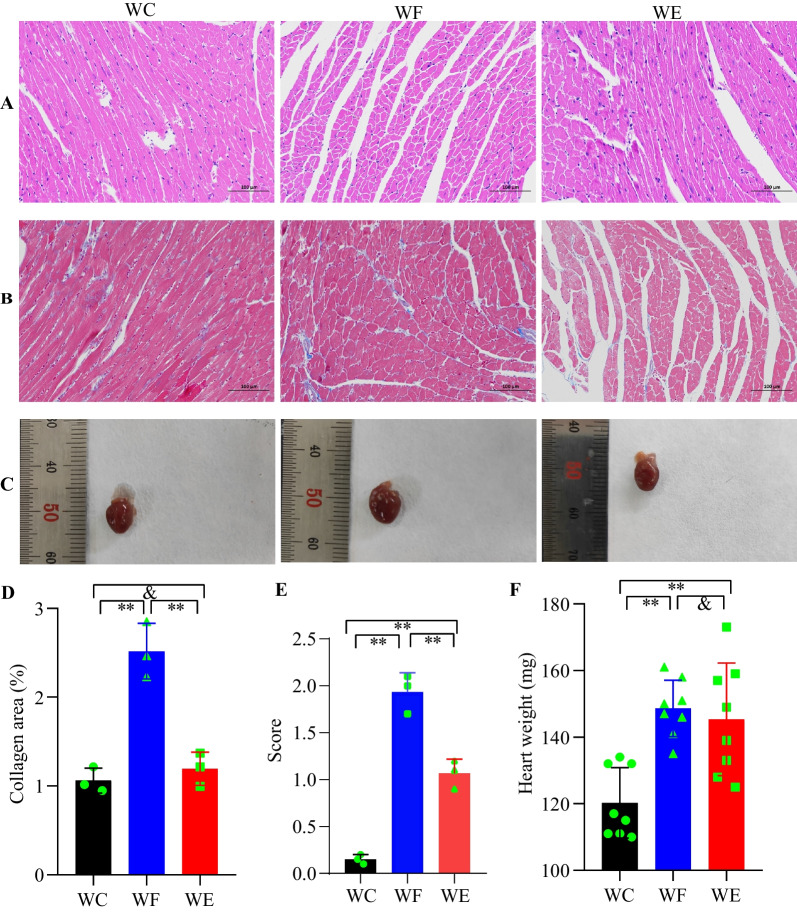
Changes in cardiac pathology with empagliflozin treatment. **A** Hematoxylin and eosin staining (200 × , *n* = 3/group). **B** Masson’s trichrome staining (200 × , *n* = 3/group). **C** Heart morphology. **D** Collagen area (%) (*n* = 3/group). **E** Semiquantitative scoring of oil red O staining of cardiac tissue. **F** Heart weight (*n* = 8/group). NS represents *P* > 0.05, **represents *P* < 0.01. Abbreviations: WC, control group; WF, high-fat diet; WE, high-fat diet + empagliflozin

HE staining showed increased myocardial interstitium with less intercellular connection, disorganized arrangement and inconsistent nuclear size in the WF group compared to the WC group, while myocardial cells in the WE group were significantly better compared to the WF group. Masson staining showed markedly increased myocardial collagen content in the WF group compared to the WC and WE groups (*P* < 0.01). Heart weight in the WF group was significantly higher than that in the WC group (*P* < 0.01), while heart weight in the WE group was slightly lower than that of the WF group but not statistically significant (*P* > 0.05) and still significantly higher than that of the WC group (*P* < 0.01). The results of oil red staining showed that cardiac tissue triacylglycerol accumulation was significantly increased in the WF group compared to the WC group (*P* < 0.01), while the triacylglycerol content was significantly lower in the WE group compared to the WF group (*P* < 0.01). The data is shown in Fig. 3.

### Ultrasonography

**Fig. 4:**
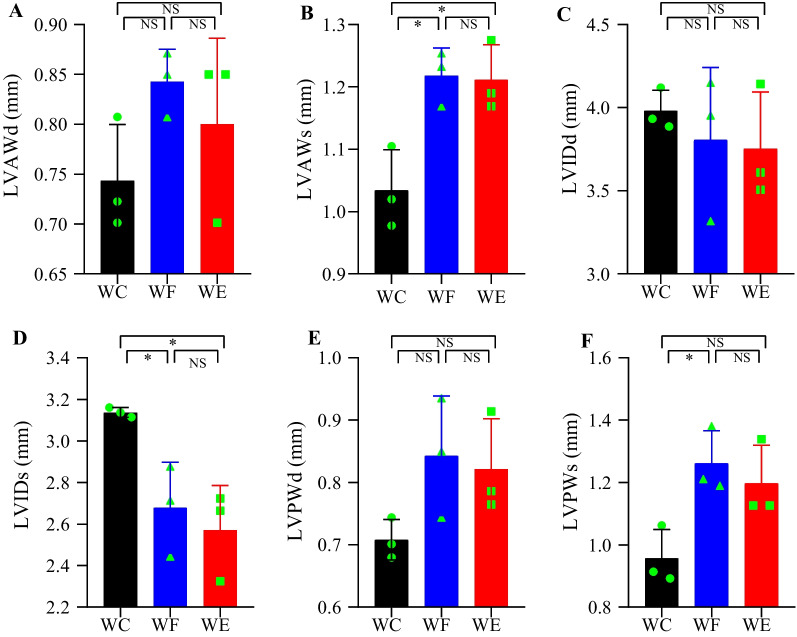
Effects of empagliflozin on cardiac function. **A** LVAWd. **B** LVAWs. **C** LVIDd. **D** LVIDs. **E** LVPWd. **F** LVPWs. *n* = 3/group. NS represents *P* > 0.05, *represents *P* < 0.05. Abbreviations: LVAWd, Left ventricular anterior wall thickness in diastole; LVAWs, Left ventricular anterior wall thickness in systole; LVIDd, Left ventricular internal dimension in diastole; LVIDs, Left ventricular internal dimension in systole; LVPWd, Left ventricular posterior wall thickness in diastole; LVPWs, Left ventricular posterior wall thickness in systole; WC, control group; WF, high-fat diet; WE, high-fat diet + empagliflozin

Cardiac M-mode ultrasound results showed that Left ventricular anterior wall thickness in systole (LVAWs) and Left ventricular posterior wall thickness in systole (LVPWs) were significantly higher in the WF group than in the WC group, whereas Left ventricular internal dimension in systole (LVIDs) were significantly lower (*P* < 0.05). Left ventricular anterior wall thickness in diastole (LVAWd) and Left ventricular posterior wall thickness in diastole (LVPWd) were also slightly increased and Left ventricular internal dimension in diastole (LVIDd) was slightly decreased in the WF group, but there was no statistically significant difference (*P* > 0.05). Mice in the WE group had reduced heart walls and enlarged inner chambers compared to the WF group, but the difference was not statistically significant (*P* > 0.05). This is consistent with previous studies that have seen that cardiac fibrosis acquired during weight gain persists after weight loss [16]. These results suggest that obesity thickens the ventricular wall and reduces the volume of the heart chambers, and that empagliflozin has a protective effect on cardiac function. Cardiac parameters are given in Fig. 4.

### The identification of differentially expressed proteins

The experiment was replicated three times in each group, and FC and *P* values were calculated based on the obtained data. When FC > 1.2 and *P* < 0.05 noted that DEPs were up-regulated, while FC < 0.83 and *P* < 0.05 indicated that DEPs were down-regulated. We identified a total of 64 DEPs in the WF/WC group, including 25 down-regulated and 39 up-regulated proteins. Compared to the WF group, empagliflozin (WE group) intervention resulted in altered expression of 83 proteins, including 21 down-regulated and 62 up-regulated proteins. To better visualize the DEPs, the volcano plot in Additional file 2 shows the DEPs.

### Key protein screening and PPI network construction

**Fig. 5:**
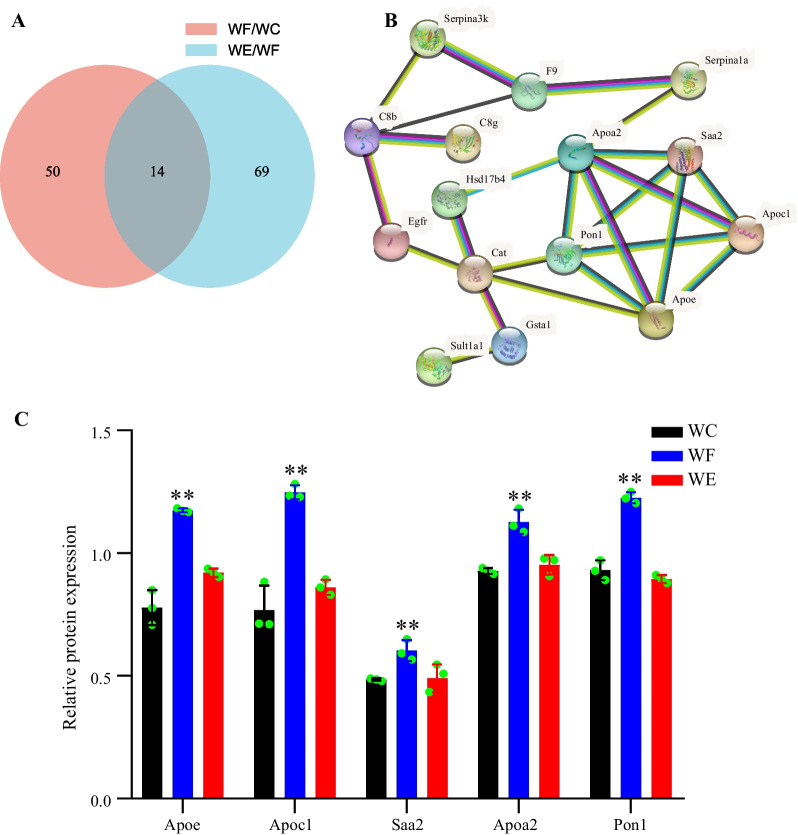
Protein expression changes and interactions. **A** Venn diagram of the common proteins of the WF/WC and WE/WF groups. **B** Protein–protein interaction network analysis. **C** Altered expression of Apoe, Apoc1, Saa2, Apoa2 and Pon1. **represents *P* < 0.01 WF versus WC and WE versus WF. Abbreviations: WC, control group; WF, high-fat diet; WE, high-fat diet + empagliflozin

By comparing WF/WC and WE/WF groups, we identified a total of 14 covariant proteins, as shown in Fig. 5A, of which Apoe, Apoc1 and Pon1 are closely associated with lipid metabolism. The PPI network of DEPs screened in the WC/WF/WE group revealed a close association between Apoe, Apoc1, Saa2, Apoa2 and Pon1. We found that Apoe, Apoc1, Apoa2 and Pon1 were mainly closely associated with cholesterol metabolism, while Saa2 was more aggregated with them and was therefore also selected as a key protein for the next study. Figure 5B shows the results of the interaction network between the proteins. Figure 5C shows our proteomic data that Apoe, Apoc1, Saa2, Apoa2 and Pon1 expression was increased in the hearts of obese mice and decreased after empagliflozin treatment (*P* < 0.01). All these proteins are closely related to lipid metabolism, and the decrease in the expression of these proteins after empagliflozin intervention suggests that empagliflozin may exert cardioprotective effects by improving lipid metabolism in obese is heart tissue.

### GO enrichment analysis

**Fig. 6:**
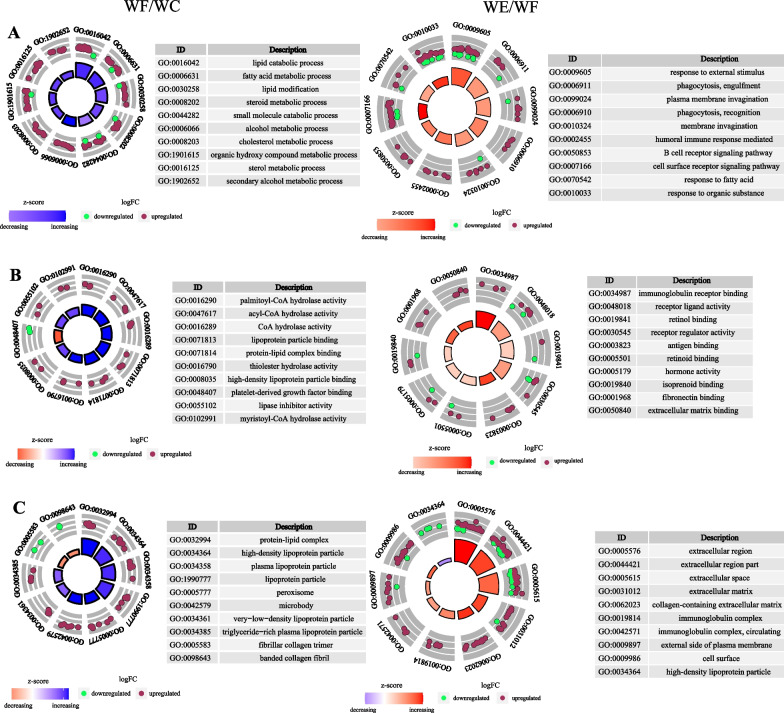
GO functional enrichment circle maps of differentially expressed proteins. **A** BP. **B** MF. **C** CC. The graph uses a circular format to show the enrichment results, with the name in the outer circle, variance cases (red for up and green for down) in the middle circle, and the z-score in the inner circle. The table data is specific information for each category. Abbreviations: WC, control group; WF, high-fat diet; WE, high-fat diet + empagliflozin; GO, Gene Ontology; BP, biological processes; MF, molecular function; CC, cell composition

GO enrichment analysis can fully demonstrate the biological and molecular functions of the proteins, so we conducted GO analysis on DEPs. In BP enrichment analysis, we found that the WF/WC and WE/WC groups were mainly enriched in the lipid metabolic process, lipid catabolic process, fatty acid metabolic process and response to fatty acid. In MF enrichment analysis, DEPs in the WF/WC group were mainly associated with palmitoyl-CoA hydrolase activity, acyl-CoA hydrolase activity, and CoA hydrolase activity, whereas in the WF/WE group, DEPs were mainly enriched in the immunoglobulin receptor binding, receptor ligand activity and retinol binding. In the WF/WC and WE/WF groups, DEPs were mainly enriched in extracellular space, extracellular region part and extracellular region in the CC analysis. Apoe, Apoc1, Saa2, Apoa2 and Pon1 were mainly associated with lipid metabolism and synthesis in GO functional enrichment analysis, and they interacted more closely with each other and were therefore selected as key proteins. Figure 6 displays the GO enrichment results of WF/WC group and WE/WF group.

### KEGG pathway analysis

**Fig. 7:**
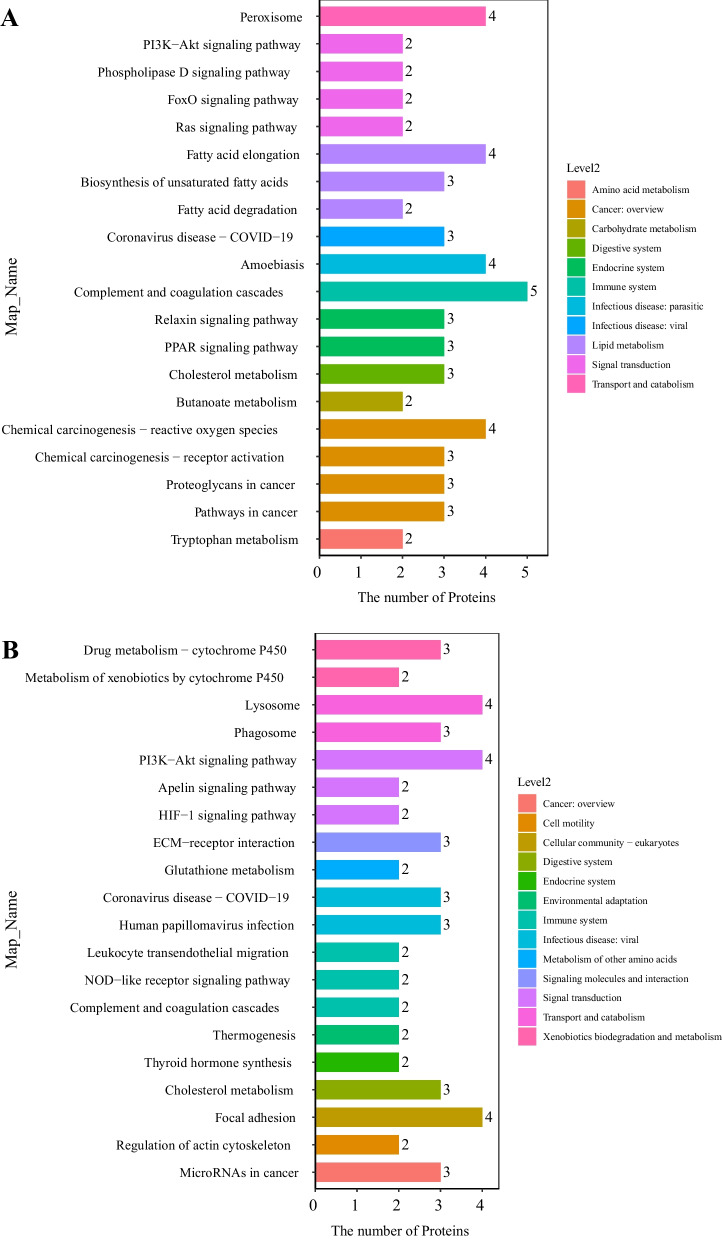
KEGG pathway enrichment analysis and construction of key protein interaction networks. **A** KEGG pathway enrichment analysis in the WF/WC group. **B** KEGG pathway enrichment analysis in the WE/WF group. Different colors are used to classify different pathways. The horizontal coordinate is the number of proteins enriched in that pathway, and the vertical coordinate is the exact name of each pathway. Abbreviations: WC, control group; WF, high-fat diet; WE, high-fat diet + empagliflozin; KEGG, Kyoto Encyclopedia of Genes and Genomes

**Fig. 8:**
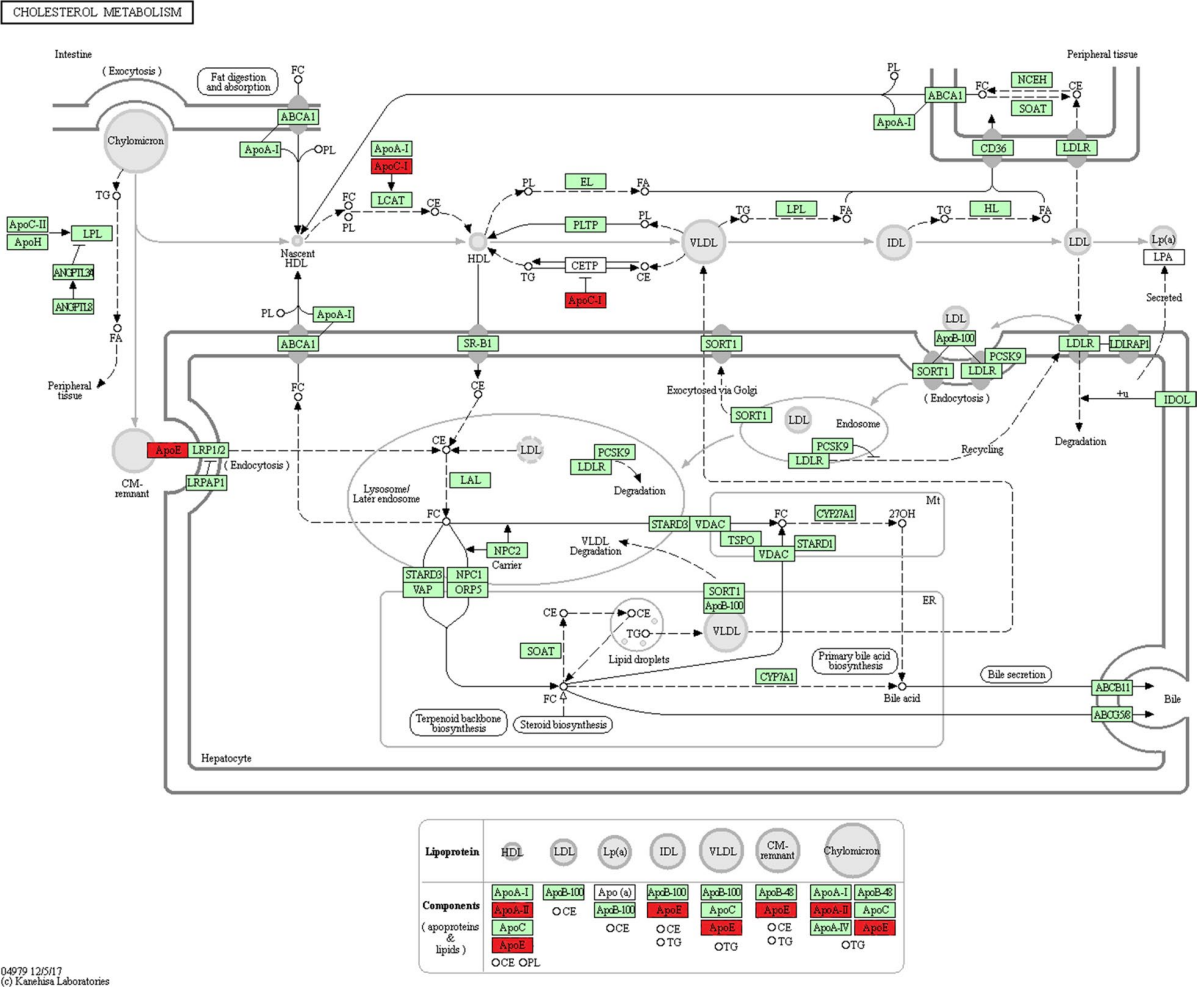
Cholesterol metabolism pathway

Pathway analysis can provide insight into the coordinated roles of proteins, so we performed KEGG pathway analysis. The results showed that in WF/WC group, DEPs were mainly associated with Complement and coagulation cascades, cholesterol metabolism and fatty acid elongation, while in WE/WF group the proteins were mainly enriched in cholesterol metabolism, ECM-receptor interaction and lysosome. Figure 7 shows the specific enriched pathway information for the WF/WC and WE/WF groups of DEPs. By pathway analysis, we found that cholesterol metabolic pathways were included in both WF/WC and WE/WF groups, where Apoe, Apoc1, and Apoa2 were co-expressed DEPs. The specific locations of Apoe, Apoc1, and Apoa2 in the cholesterol metabolism pathway are indicated in Fig. 8.

## Discussion

SGLT-2i is a new class of hypoglycemic medicines whose major mechanism of action is to selectively inhibit sodium and glucose reabsorption from the proximal renal tubule and increase glucose excretion in the urine, lowering blood glucose levels [21, 22]. Empagliflozin is a member of this class. Empagliflozin considerably lowers the occurrence of cardiovascular events in individuals with diabetes mellitus and heart failure, according to clinical investigations [23, 24]. Empagliflozin is thought to have a cardiovascular preventive effect through improving glycerolipid metabolism and insulin resistance [25]. This study also found that treating obese mice with empagliflozin improved glycerolipid metabolism. HDL-C was significantly higher in obese mice and lowered following empagliflozin treatment, which is currently assumed to be due to an increase in total lipids, and it has been hypothesized that its elevation is unrelated to function. Some investigations [26, 27] have proven empagliflozin's cardioprotective efficacy in obese people without concomitant diabetes. Empagliflozin did not significantly improve obesity-induced cardiac hypertrophy in our study, which could be due to the short duration of administration and low dose of the drug, but it did reduce myocardial collagen content, and inhibit myocardial remodeling, which is consistent with previous findings [28].

Weight loss greatly alleviates the aberrant protein expression generated by obesity, resulting in cardioprotective effects, according to proteomics studies [16, 29]. The current study found that obese mice's body weight reduced dramatically following treatment with empagliflozin, but it's still unclear if the cardioprotective effect of empagliflozin on obesity is mediated by weight loss or by other mechanisms. It has been postulated that Sestrin2-mediated AMPK-mTOR signaling is important in empagliflozin's cardioprotective properties [8]. Apoe, Apoc1, Saa2, Apoa2, and Pon1 (all implicated in cholesterol metabolism) were found to change considerably after empagliflozin treatment, but not in the presence of weight loss [16]. In addition, the main mechanism of action of empagliflozin is the inhibition of renal reuptake of glucose, the HFD provided in this study is triacylglycerol rich and it is unclear whether the reduction in body weight in mice is associated with enhanced β-oxidation of fat. The present study showed that empagliflozin did improve lipid metabolism in obese mice, which provides a new idea for the next study. As a result, it's plausible to assume that empagliflozin's cardioprotective impact isn't just due to weight loss.

Apolipoprotein E (Apoe) is a 34-kDa glycoprotein that can be released by a variety of cells. Its primary function is lipid metabolism, but it has also been implicated in the development of cardiovascular disease and obesity in recent research [30, 31]. ApoE2, apoE3, and apoE4 are the three primary isoforms of this apolipoprotein. Apoe is thought to play a role in the development of atherosclerosis since E4 gene carriers have higher cholesterol levels [32]. The connection between Apoe and atherosclerosis, on the other hand, has been shown to be independent of lipid alterations [33]. Furthermore, during atherosclerosis, macrophages in the artery wall might release Apoe and suppress vascular cell adhesion molecule 1 (VCAM-1) secretion, thus contributing to the inflammatory response [31]. Increased levels of blood Apoe have also been linked to metabolic syndrome (obesity, dyslipidemia, and insulin resistance) in clinical investigations [32, 34]. The current study found higher levels of Apoe expression in cardiac tissues in obese people and lower levels following empagliflozin treatment, indicating that lipid metabolism abnormalities are improving.

Apolipoprotein C1 (Apoc1) is the smallest apolipoprotein in the family and is involved in lipid metabolism [35]. Apoc1 has now been linked to lung cancer, kidney cancer, and gastrointestinal malignancies in a number of studies [36–38]. Alzheimer's disease, diabetes, and atherosclerosis have all been associated to the progression of Apoc1 [39, 40]. Transgenic mouse investigations have revealed that Apoc1 inhibits lipoprotein lipase (LPL) function, causing aberrant lipid elevations, and that Apoc1 transgenic animals significantly raise inflammation levels in vivo [41, 42]. Apoc1 has been linked to the production of foam cells in a growing number of studies, suggesting that it has an atherogenic effect. Furthermore, investigations in mice overexpressing Apoc1 have shown that Apoc1 is linked to liver fibrosis and insulin resistance [43, 44]. There are, however, less investigations on Apoc1 and obesity-related heart damage. Our discovery that empagliflozin reduces Apoc1 expression, which could be linked to cardioprotective benefits, has to be investigated further.

The acute-phase response protein serum amyloid A (SAA) has two members: Saa1 and Saa2, and serum levels rise fast in response to trauma, infection, and other stimuli [45]. Obesity-related increases in SAA production by adipose tissue have been linked to increased inflammation in the body and are closely linked to obesity and its consequences (atherosclerosis and insulin resistance) [46]. It is feasible to conclude that Saa2 causes heart damage mostly due to higher levels of inflammation and lipid metabolic abnormalities, and that inhibiting Saa2 expression has a cardioprotective impact.

Apolipoprotein A-II (Apoa2) is a key component of HDL and belongs to the apolipoprotein family. Recent research has discovered a substantial link between Apoa2 and lipid metabolism as well as insulin resistance. Apoa2 and insulin resistance are negatively correlated in both animal and human studies [47, 48]. The link between Apoa2 and atherosclerosis, on the other hand, remains unclear. Some research has found a link between residual atherosclerosis and Apoa2, whereas others have found Apoa2 to be an atherogenic factor [49, 50]. Obesity increased Apoa2 expression in the current study, and it decreased following the empagliflozin intervention, but more research is needed to evaluate whether empagliflozin has cardioprotective effects by changing Apoa2 expression. Unlike Apoa2, paraoxonase-1 (Pon1) has been linked to oxidative stress and inflammatory disorders and has been deemed a cardiovascular preventive factor. The increased level of Pon1 expression in cardiac tissue during obesity in this study indicates an increase in local inflammation in cardiac tissue, necessitating more Pon1 to maintain normal myocardial function, whereas the decreased level of Pon1 expression after empagliflozin administration indicates a decrease in cardiac tissue inflammation.

There are some shortcomings in this study. First, our study showed that empagliflozin significantly reduced body weight in obese mice, and whether its exerting cardioprotective effect is achieved by reducing body weight still needs to be confirmed by further studies. Secondly, normal control mice were not treated with empagliflozin intervention to study the independent effects of the drug. Finally, we did not perform functional measurements of the heart. Although the study has some limitations, our findings provide a good basis for future studies and provide new ideas for the clinical use of empagliflozin.

## Conclusions

Empagliflozin ameliorates disorders of glucolipid metabolism and reduces cardiac collagen content in obese mice fed a high-fat diet. Apoe, Apoc1, Saa2, Apoa2 and Pon1 and their involvement in cholesterol metabolism may be the mechanisms underlying the cardioprotective effects of empagliflozin.

## Supplementary Information

**Additional file 1**: The specific composition of the diet.

**Additional file 2**: Volcano plots of differentially expressed proteins. Volcano plots of differentially expressed proteins were in the WF/WC (A) and WE/WF (B) groups, respectively. Each dot is a protein. Red represents up-regulated protein expression (Fold Change > 1.2 and *P*-value < 0.05); green represents down-regulated protein expression (Fold Change < 0.83 and *P*-value < 0.05). The horizontal coordinate is a logarithmic transformation of the Fold Change with a base of 2, and the vertical coordinate is a logarithmic transformation of the *P*-value with a base of 10. Abbreviations: WC, control group; WF, high-fat diet; WE, high-fat diet + empagliflozin.
