# Supplementary material for: Effect of high-fat diet and empagliflozin on cardiac proteins in mice
Source: Nutr Metab (Lond). 2022 Oct 14;19:69. doi: 10.1186/s12986-022-00705-0 (PMC9563173; doi:10.1186/s12986-022-00705-0)
Supplement: Supplementary file 1 — Additional file 1: The specific composition of the diet. [file 12986_2022_705_MOESM1_ESM.docx]

| Composition | g | Kcal |
| --- | --- | --- |
| Tyrosine | 258.45 | 1033.8 |
| Cystine | 3.88 | 15.52 |
| Maltodextrin | 161.53 | 646.12 |
| Sucrose | 88.91 | 355.64 |
| Cellulose | 64.61 | 0 |
| Soybean Oil | 32.31 | 290.79 |
| Lard | 316.6 | 2849.4 |
| Mineral mixture M1002 | 12.92 | 0 |
| Calcium hydrogen phosphate | 16.8 | 0 |
| Calcium carbonate | 7.11 | 0 |
| Potassium citrate | 21.32 | 0 |
| Vitamin Blend V1001 | 12.92 | 51.68 |
| Hydrocholine bitartrate | 2.58 | 0 |
| Food blue dye | 0.065 | 0 |
| total | 1000 | 5242.95 |

Nutritional composition of high-fat chow

Nutritional composition of standard mouse chow

| Main ingredients |  | Nutrient content values |  |
| --- | --- | --- | --- |
| Protein Source | Soybean meal, Fish Meal | Moisture% | ≤10 |
| Fat source | Vegetable oil | Crude protein% | ≥18 |
| Fiber Source | Bran | Crude fat% | ≥4 |
| Carbohydrate source | Corn, secondary flour | Crude fiber% | ≤5 |
| Vitamins | VA, VD, VE, VB1, VB2, VB6, Pantothenic acid | Crude Ash% | ≤8 |
| Minerals | Calcium bicarbonate, Stone powder, Iron, Copper, Manganese, Zinc, etc | Calcium% | 1-1.8 |
|  |  | Phosphorus% | 0.6-1.2 |
