# Supplementary figures and images for: Effect of high-fat diet and empagliflozin on cardiac proteins in mice
Source: Nutr Metab (Lond). 2022 Oct 14;19:69. doi: 10.1186/s12986-022-00705-0 (PMC9563173; doi:10.1186/s12986-022-00705-0)

**A**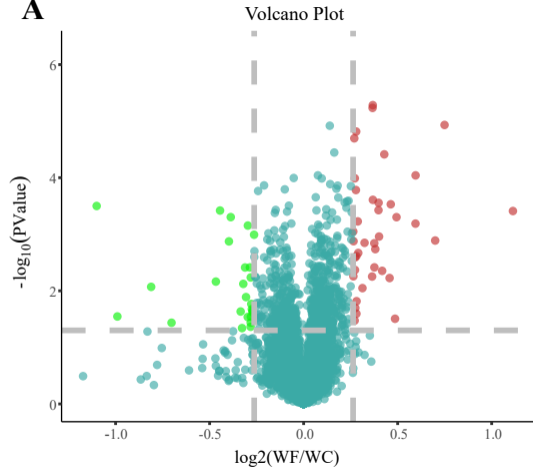**B**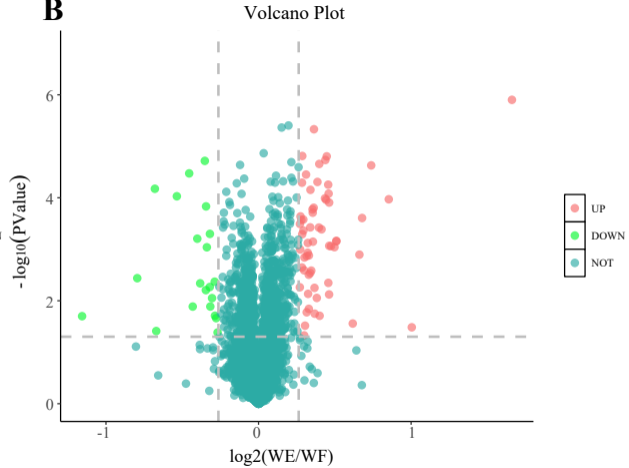

Supplement: Supplementary file 2 — Additional file 2: Volcano plots of differentially expressed proteins. Volcano plots of differentially expressed proteins were in the WF/WC (A) and WE/WF (B) groups, respectively. Each dot is a protein. Red represents up-regulated protein expression (Fold Change > 1.2 and P-value < 0.05); green represents down-regulated protein expression (Fold Change < 0.83 and P-value < 0.05). The horizontal coordinate is a logarithmic transformation of the Fold Change with a base of 2, and the vertical coordinate is a logarithmic transformation of the P-value with a base of 10. Abbreviations: WC, control group; WF, high-fat diet; WE, high-fat diet + empagliflozin. [file 12986_2022_705_MOESM2_ESM.pdf]
